# Reviewer acknowledgement 2014

**DOI:** 10.1186/s40463-015-0054-x

**Published:** 2015-02-11

**Authors:** Hadi Seikaly, Erin Wright

**Affiliations:** University of Alberta, Alberta, Canada

## Abstract

The Editors of *Journal of Otolaryngology - Head & Neck Surgery* would like to thank all our reviewers who have contributed to the journal in Volume 43 (2014).

**Peter Adamson**

Canada

**Sumit Agrawal**

Canada

**Yaser Alrajhi**

Canada

**Jennifer Anderson**

Canada

**Don Anderson**

Canada

**Kal Ansari**

Canada

**Margaret Aron**

Canada

**Rick Balys**

Canada

**Sarfaraz Banglawala**

Canada

**Vincent Biron**

Canada

**Brian Blakley**

Canada

**Nikolas Blevins**

USA

**James Bonaparte**

Canada

**Michael Brandt**

Canada

**Martin Bullock**

Canada

**Marie Bussieres**

Canada

**Neil Chadha**

Canada

**Yvonne Chan**

Canada

**Shamir Chandarana**

Canada

**Justin Chau**

Canada

**Gabriela Constantinescu**

Canada

**David Cote**

Canada

**Sam Daniel**

Canada

**Chris Diamond**

Canada

**Dominique Dorion**

Canada

**Joseph Dort**

Canada

**Jaymi Dumper**

Canada

**Hamdy El-Hakim**

Canada

**Danny Enepekides**

Canada

**Jeremy Freeman**

Canada

**Kevin Fung**

Canada

**Richard Gall**

Canada

**Nahla Gomaa**

Canada

**Menachem Gross**

Israel

**Trevor Hartl**

Canada

**Eric Henry**

Canada

**Michael Hier**

Canada

**Omar Hilmi**

United Kingdom

**Allan Ho**

Canada

**Jordan Hochman**

Canada

**Paul Hong**

Canada

**Caroline Jeffery**

Canada

**Liane Johnson**

Canada

**Jodi Jones**

Canada

**Paul Kerr**

Canada

**Safeena Kherani**

Canada

**Haytham Kubba**

United Kingdom

**François Lavigne**

Canada

**Jane Lea**

Canada

**John Lee**

Canada

**Boyd Lee**

Canada

**Darren Leitao**

Canada

**Wei Li**

USA

**Vincent Lin**

Canada

**Kristian Macdonald**

Canada

**Emad Massoud**

Canada

**Laurie McLean**

Canada

**Deepak Mehta**

USA

**Adrian Mendez**

Canada

**Paul Mick**

Canada

**Alex Mlynarek**

Canada

**Corey Moore**

Canada

**Sharon Morong**

Canada

**David Morris**

Canada

**Lily HP Nguyen**

Canada

**Anthony Nichols**

Canada

**Desmond Nunez**

Canada

**Dan O'Connell**

Canada

**Heather Osborn**

Canada

**Flordeliz Osler**

Canada

**Richard Payne**

Canada

**Evan Propst**

Canada

**Keith Richardson**

Canada

**Matthew Rigby**

Canada

**Brian Rotenberg**

Canada

**Kathryn Roth**

Canada

**Luke Rudmik**

Canada

**Issam Saliba**

Canada

**Mark Samaha**

Canada

**Julian Savage**

Canada

**David Schramm**

Canada

**Anil Sharma**

Canada

**Praby Singh**

USA

**Sue Rene Soon**

Singapore

**Leigh Sowerby**

Canada

**Peter Spafford**

Canada

**Chris Szeto**

Canada

**George Tavartkiladze**

Russia

**Mark Taylor**

Canada

**Jamie Tibbo**

Canada

**Jonathan Trites**

Canada

**Trina Uwiera**

Canada

**Jean-Philippe Vaccani**

Canada

**Allan Vescan**

Canada

**Scott Weichenthal**

Canada

**Manisha Witmans**

Canada

**Anthony Zeitouni**

Canada

